# Recipe

**Published:** 1853-04

**Authors:** Jno. McClure


### RECIPE.

The following recipe for diseases of the gums when the alveolar processes are wasting away, is given us in a letter by Dr. McClure of Carrolton Mississippi. If it does half which is attributed to it, it is worth its weight in gold.

Ed.

Take sulphate of Iron, (common copperas,) calcine it in a strong heat for three hours at least or until it becomes a dark olive brown, it is then a tasteless inodorous substance called crous mastus, reduce it to a fine powder, mix it with water or alcohol. It is a heavy drug; alcohol has but little action upon it; just before useing it should be shook up; with an instrument—a small point made of tough wood, and a little cotton, manage to get this drug in contact with the diseased alveolus. The tartar must be removed; the gums made to bleed freely, apply the medicine once a day at least, it will take from two to six weeks to effect a cure. But it is a specific. Try it, and let me hear from you. It will even arrest caries of the teeth. If I should ever hear from you, and you believe it to be good, I will then tell you what induced me to use it.

Yours, Respectfully, JNO. McCLURE.

